# Sensing a revolution

**DOI:** 10.15252/msb.20166873

**Published:** 2016-04-26

**Authors:** Lars M Steinmetz, Allan Jones

**Affiliations:** ^1^ Stanford Genome Technology CenterPalo AltoCAUSA; ^2^ Department of GeneticsStanford University School of MedicineStanfordCAUSA; ^3^ European Molecular Biology Laboratory (EMBL)Genome Biology UnitHeidelbergGermany

**Keywords:** Systems Medicine

## Abstract

New fully integrated biosensors that monitor molecular and physiological parameters throughout our bodies are set to revolutionize medicine and personalized healthcare.

In the movie SPECTRE, MI6 scientists equip James Bond with the usual collection of gadgets, including the obligatory high‐end car and exploding watch. James also receives an injection of “smart blood”—a nanoscale sensor that continuously tracks the carrier's location based on a GPS signal emitted from inside the body. While this is only science fiction, the concept is actually not that far from reality. Biodegradable sensors are being developed that decompose into nontoxic components once they are no longer needed, with potential applications including disease diagnosis and targeted drug delivery. We anticipate that in the coming years, revolutionary devices will be built at the interface between electrical engineering, material science, and biomedicine that will measure, process, and integrate hundreds of physiological and environmental parameters in real time.

Today, fashionable fitness trackers and smart watches that monitor our physical activity, pulse rates, and sleep patterns are growing in popularity, generating a growing and highly competitive market expected to be worth around $25 billion by 2019 (Lamkin, 2015). While consumers currently use such devices to track their daily exercise regimen and optimize their fitness profile, the next generation of biosensors will be able to monitor complex physiological parameters, with potential applications ranging from sports management to healthcare. These biosensors will be integrated into wearable, implantable, and even edible devices to monitor multiple aspects of our physiology by sampling different bodily fluids, including blood, sweat, tears, and saliva (Bandodkar & Wang, 2014). Multidimensional personal biological profiles will combine data from numerous types of biomarkers, including metabolites, electrolytes, nucleic acids, proteins, and whole cells, with measured key environmental variables such as temperature, food intake, microbial load, or air pollutants. Processing such data from hundreds of thousands of individuals will enable the development of training algorithms that detect deviations from an individual's healthy profile and associate the altered state with observed disease outcomes. Ultimately, we envision that the continuous collection of integrated personalized molecular and environmental profiles will assist the early detection and even prediction of disease onset, before overt symptoms arise.

## Portable healthcare devices at the point of living

**Figure 1:**
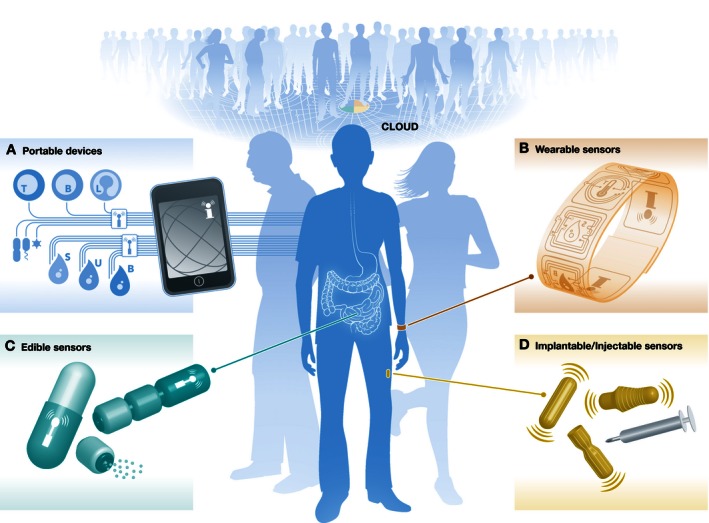
**Integrated biosensors to monitor personalized health.** Multiple sensors are spread around the body to continuously collect data. (A) Portable devices for individuals to independently monitor health parameters at the point of living (T: T‐cells; B: B‐cells; L: other leukocytes; S: saliva, U: urine, B: blood). (B) Noninvasive wearable sensors to continuously monitor physiological parameters from on the body surface. (C) Edible sensors to measure molecular parameters and vital signs from inside the digestive tract. (D) Implantable or injectable sensors to monitor molecular parameters directly on a tissue or in the bloodstream.

Easy‐to‐use, portable medical devices that monitor health parameters from small amounts of bodily fluids have the potential to bring healthcare directly to the individual's point of living, even in remote or underdeveloped settings. Ideally, these devices should be non‐ or minimally invasive and capable of measuring multiplexed biomarkers in a simple, rapid, and reliable manner when operated by the individuals themselves. Wireless transmission of the data to healthcare providers located further afield would enable remote monitoring and facilitate access to the medical expertise necessary to make sound treatment decisions (Fig 1A). Our group recently developed a cost‐effective magnetic levitation platform that sorts and quantifies different cell types, including bacteria and circulating tumor cells, based on their unique density profiles (Durmus *et al*, 2015). The platform can be combined with a smartphone, using its high‐resolution camera to acquire images of cells before the data is processed in the cloud (Baday *et al*, 2015). In the future, similar fully integrated, highly sensitive devices that analyze human samples could be used to detect the emergence of infectious diseases or monitor antibiotic resistance, also in developing countries in which there is limited access to healthcare. In this respect, keeping sample processing as simple as possible is key to ensuring participant compliance. Such portable technology can be deployed widely as the assays occur at the micro‐ or nanoscale, production costs are low, and the devices do not require extensive external laboratory infrastructure. As they are adopted globally, these technologies will also contribute to the collection of large data sets at the population level to improve computational models.

## Wearable devices for continuous molecular profiling

Unlike portable platforms, wearable biosensors such as the popular fitness trackers gather data autonomously without requiring any input from the user (Fig 1B). By shrinking the size of electrical circuits and developing flexible materials that adapt to movement and swelling, bioengineers are making it possible to exploit new data sources throughout our bodies. Google is prototyping contact lenses that incorporate optical sensors to measure glucose levels from tear fluid (Yao *et al*, 2012; Bandodkar & Wang, 2014). If such technology can be made reliable and affordable, it could simplify the lives of diabetic patients by providing a noninvasive way to alert them as soon as systemic glucose levels pass a dangerous threshold. Other novel biosensors measure physiological parameters from sweat, an underexplored and highly complex mixture of molecules. Scientists at UC Berkley and the Stanford Genome Technology Center, for instance, have built a flexible plastic‐based biosensor that simultaneously measures body temperature, metabolites (glucose and lactate), and electrolytes (sodium and potassium) in sweat directly on the skin (Gao *et al*, 2016). The device automatically calibrates itself according to temperature and transmits data to a cloud server in real time. Initial data generated with this device indicate that an increase in sodium levels serves as a reliable biomarker for impending dehydration. Given that sweat could be such a rich source of physiological information, we anticipate that it will be exploited for many other diagnostic applications. For example, inference of blood glucose levels based on sweat glucose concentrations is currently being explored. In general, we expect wearable biosensors to play a more and more important role in the primary healthcare setting due to their noninvasiveness and autonomy, which enable continuous longitudinal monitoring of individuals’ physiology. In particular, dynamic information concerning the stability, variability, or change rate of key physiological and metabolic parameters in response to challenges is likely to provide unique insights into the state of physiological systems in health and reliably predict disease. Noninvasive monitoring of hundreds to thousands of different molecules would, for example, benefit the elderly and patients suffering from chronic diseases. Systems that track their vital signs or even deliver targeted doses of medication according to learnt patterns would raise the standard of care to the next level.

## Beyond wearables: implantable and edible biosensors

The next logical step in biosensor technology is to develop devices that go beneath the skin. This can be achieved with implants, via the bloodstream or by ingestion into the digestive tract. In all three cases, bioengineers are confronted with a new series of technological challenges. For one, all components of the sensors, including the circuitry and wireless transmitters, must consist of advanced biocompatible or biodegradable materials that do not cause adverse reactions or inflammation in the host. Also, to function over long periods, these biosensors need to have renewable and safe energy sources. Innovative solutions to these problems include utilizing the mechanical energy generated by body movements or leveraging the motions of the heart, lung, or diaphragm (Dagdeviren *et al*, 2015; Gibney, 2015). Alternatively, biodegradable batteries that disintegrate into nontoxic components over time could be used as energy sources to power temporary devices. In this respect, engineers recently developed biocompatible melanin electrodes for sodium ion storage that produce enough energy to power implantable devices like pacemakers (Kim *et al*, 2013).

As the technical barriers are overcome one by one—and progress is rapid—such biosensors will provide access to a whole new range of biological information. Edible sensors, for example, represent an attractive, nonobtrusive approach to measure internal vital signs (Fig 1C). In this respect, MIT scientists recently tested a device to monitor the heart rate and respiratory rate using sound waves in the gastrointestinal tract of pigs (Traverso *et al*, 2015). Also, we envision that biosensors capable of continuously monitoring the abundance of tissue‐specific proteins in the bloodstream will be used to detect the onset of diseases like cancer and determine the affected organs reliably (Fig 1D).

In conclusion, the development of cost‐effective biosensor technologies that continuously sense multiple parameters is progressing at an exciting pace and will become a key component of the personalized health revolution. Analysis and interpretation of the huge data streams produced by networks of integrated molecular and environmental biosensors across millions of individuals will represent a formidable challenge of both technical and regulatory nature. Successfully developing these approaches will be necessary to make precision medicine a reality and will open new avenues in detecting, predicting, and preventing disease. We are only at the beginning of this revolution, but already now it is clear that the real potential of “smart blood” will go far beyond what Q had designed to save her Majesty's agents.

## Conflict of interest

L.M.S. is President of the Scientific Advisory Board and a founder of Sophia Genetics, Switzerland. This company analyzes clinical genomics data.
